# A Patient with an Unusual Cause Right Lower Quadrant Pain and Vomiting: Pyelonephritis of an Ectopic Right Kidney Masquerading as Acute Appendicitis

**DOI:** 10.1155/2009/638501

**Published:** 2010-02-04

**Authors:** Michele N. Lossius, Carlos E. Araya, Dwayne D. Henry, Richard E. Neiberger

**Affiliations:** ^1^Department of Pediatrics, College of Medicine, University of Florida, Gainesville, FL 32608, USA; ^2^Department of Pediatrics, College of Medicine, University of Oklahoma Health Science Center, Oklahoma City, OK 73104, USA

## Abstract

An adolescent female presented with one day of abdominal pain and clinical findings of acute appendicitis. CT scan revealed an ectopic right kidney with changes of acute pyelonephritis. This paper underscores the importance of imaging the right pelvis prior to surgical intervention in suspected cases of acute appendicitis in children.

## 1. Introduction

The diagnosis of acute pyelonephritis can frequently be made based on the patient's history, physical examination, and simple laboratory studies obtained at the time of the initial clinical encounter. However, in children and adolescents, making the diagnosis can be difficult if the presentation is atypical and mimics other conditions. Delay in the diagnosis of acute pyelonephritis can lead to renal scarring which in turn may be associated with hypertension and chronic renal failure. 

 Abdominal pain is a common complaint in children and adolescents. Most physicians relate right lower quadrant abdominal pain and fever to acute appendicitis. However, the differential diagnosis with this clinical presentation is broad and can vary based on the age and gender of the patient. In patients with an ectopic kidney, acute pyelonephritis will present with abdominal pain which may simulate acute appendicitis. Hence, abdominal imaging of children with suspected acute appendicitis prior to surgical intervention is important since it will decrease negative appendectomy rates and avoid unnecessary surgery in these cases. 

 Herein, we report a case of renal ectopia and pyelonephritis presenting as right lower quadrant abdominal pain and suspected appendicitis in an adolescent female.

## 2. Case Report

The patient is a previously healthy sixteen-year-old female with acute onset of right lower quadrant abdominal pain associated with nausea and vomiting that began one day prior to admission. She denied illicit drug use, sexual intercourse, or trauma to the area. Review of systems provided no history of fever, cough, chest pain, dysuria, urgency, frequency, hematuria, or menstrual abnormality. The family history was noncontributory. 

 In the emergency department she was afebrile but had a heart rate of 109 beats per minute and a blood pressure of 101/52 mm Hg. On examination, she was alert and interactive. The abdomen was nondistended, and she had normal active bowel sounds. However, she was tender to palpitation in the right lower quadrant with rebound tenderness and voluntary guarding. There was no costovertebral angle tenderness. The remainder of the physical exam was normal. 

 Laboratory studies showed a hemoglobin level of 12 g/dL; platelet count 273 million/L; Leukocyte count 28.1 thousand/mL with predominately neutrophils (91%). Serum chemistry revealed mild hyponatremia (131 mEq/L) with otherwise normal electrolytes, liver, and renal function. The urinalysis had greater than 100 white blood cells per high power field and moderate bacteria without hematuria or cast formation. A urine pregnancy test was negative. 

**Figure 1:**
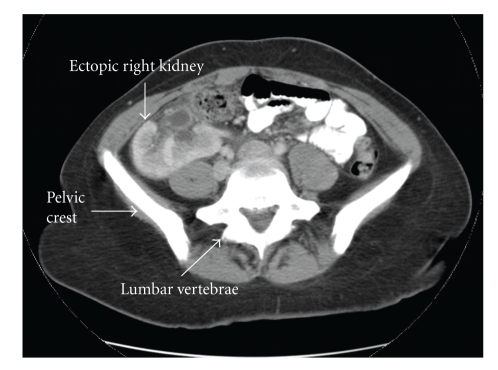
Right Lumbar Kidney. Transverse view of the pelvis reveals the ectopic right kidney. The collecting system is anterior from incomplete rotation of the kidney.

**Figure 2:**
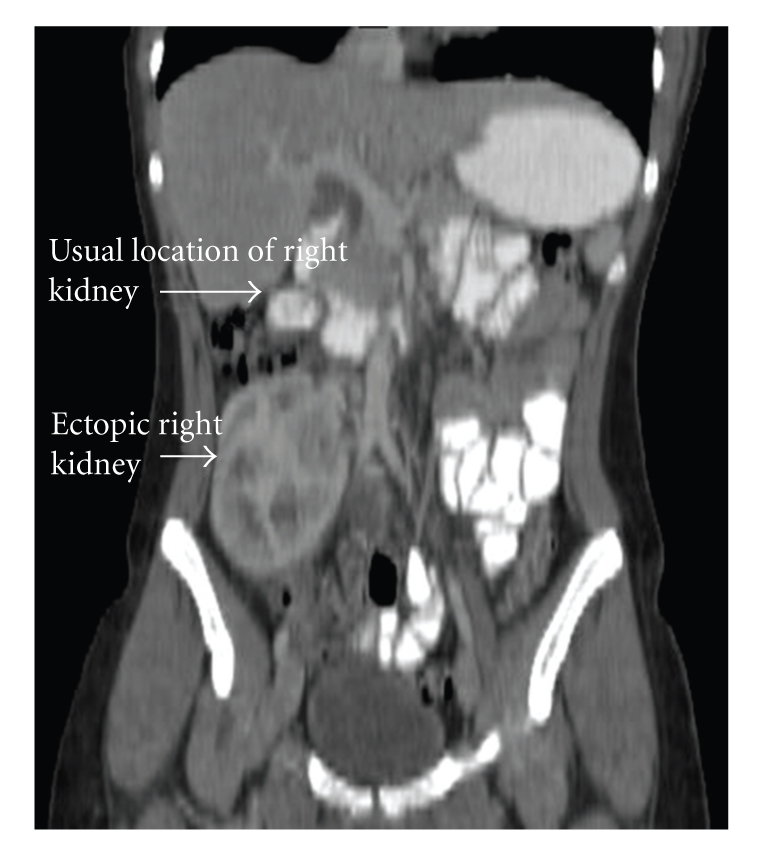
Coronal view right ectopic kidney. The right kidney rests in the iliac fossa at the level of the aortic bifurcation.

 In the emergency room a CT scan of the abdomen was done. The study showed a normal left kidney and an ectopic right-sided kidney localized in the right lower quadrant of the abdomen (Figures 1, 2). The CT scan showed a striated right renogram consistent with acute pyelonephritis. No other uro-genital anomalies were identified.

Due to persistent hypotension and concern for septic shock, she was given three-one liter boluses of normal saline and was admitted to the Pediatric Intensive Care Unit. Initial antibiotic therapy consisted of ampicillin and gentamicin. A norepinephrine infusion was administered for hypotension during the first 24 hours. Subsequently, the urine culture grew *Escherichia coli*. 

 A voiding cystouretrogram (VCUG) was normal without vesicoureteral reflux (VUR) into the ectopic kidney. After seven days of intravenous antibiotics, she was discharged home on oral ciprofloxacin and had an otherwise uneventful recovery.

## 3. Discussion

 Ectopic kidneys result from anomalies of ascent and can be found in the pelvis, iliac, abdominal, thoracic, or contra-lateral positions. Problems in the ureteral bud and metanephric tissue as well as genetic abnormalities have been implicated in renal ectopia. The prevalence of pelvic kidney is probably between 1/500 and 1/3000, with the left side more commonly affected [1, 2]. Bilateral renal ectopia is rarely encountered. 

 Several complications may occur with pelvic kidney [3]. The affected kidney is usually hypoplastic and the collecting system anterior as a result of incomplete rotation. VUR, can also occur in up to 30% of pelvic kidneys. Over half of ectopic kidneys have a dilated collecting system with hydronephrosis as a result of the malrotation alone, high grade VUR or obstruction at the level of the ureteropelvic or ureterovesical junction. Most individuals with ectopic kidneys are asymptomatic; however, the most common complications occur from obstruction, infection, and urolithiasis. 

 Individuals with renal ectopia have an increased incidence of other genital anomalies. Females have a higher incidence of unicornuate or bicornuate uterus as well as vaginal abnormalities. In males, cryptorchidism, hypospadias, or urethral duplications may be seen. In most patients with an ectopic kidney, the contralateral kidney is normal. The risk of malignancy in the ectopic kidney is not increased. 

 A literature search for the last twenty-five years reveals only two other published cases of ectopic kidney pyelonephritis [4, 5]. The two reports represent a total of 4 cases in which ectopic kidney pyelonephritis simulated acute appendicitis or bowel perforation. In our patient, the initial clinical diagnosis was acute appendicitis. However, in children and adolescents, an accurate clinical diagnosis of acute appendicitis is difficult to make and children undergoing emergency appendectomy have a normal appendix removed in 15% to 40% of cases [6]. 

 This paper highlights the importance of imaging the right pelvis prior to surgical intervention in suspected cases of acute appendicitis as well as considering acute pyelonephritis of an ectopic kidney in the differential diagnosis of lower quadrant abdominal pain.

##  Financial Disclosure

No disclosure to report for any author.

##  Proprietary Statement

The authors have no proprietary interest.
